# Cardiac magnetic resonance for prophylactic implantable-cardioverter defibrillator therapy international study: prognostic value of cardiac magnetic resonance-derived right ventricular parameters substudy

**DOI:** 10.1093/ehjci/jeac124

**Published:** 2022-07-06

**Authors:** Subhi J Al’Aref, Ahmed M Altibi, Abdallah Malkawi, Munthir Mansour, Lohendran Baskaran, Ahmad Masri, Hind Rahmouni, Raffaele Abete, Daniele Andreini, Giovanni Aquaro, Andrea Barison, Jan Bogaert, Giovanni Camastra, Samuela Carigi, Nazario Carrabba, Grazia Casavecchia, Stefano Censi, Gloria Cicala, Edoardo Conte, Carlo N De Cecco, Manuel De Lazzari, Gabriella Di Giovine, Mauro Di Roma, Monica Dobrovie, Marta Focardi, Nicola Gaibazzi, Annalaura Gismondi, Matteo Gravina, Marco Guglielmo, Chiara Lanzillo, Massimo Lombardi, Valentina Lorenzoni, Jordi Lozano-Torres, Davide Margonato, Chiara Martini, Francesca Marzo, Piergiorgio Masci, Ambra Masi, Riccardo Memeo, Claudio Moro, Saima Mushtaq, Alberto Nese, Alessandro Palumbo, Anne Giulia Pavon, Patrizia Pedrotti, Mauro Pepi, Martina Perazzolo Marra, Silvia Pica, Silvia Pradella, Cristina Presicci, Mark G Rabbat, Claudia Raineri, Jose’ F Rodriguez-Palomares, Stefano Sbarbati, U Joseph Schoepf, Angelo Squeri, Nicola Sverzellati, Rolf Symons, Emily Tat, Mauro Timpani, Giancarlo Todiere, Adele Valentini, Akos Varga-Szemes, Alessandra Volpe, Laura Fusini, Andrea Igoren Guaricci, Jurg Schwitter, Gianluca Pontone

**Affiliations:** Department of Medicine, Division of Cardiology, University of Arkansas for Medical Sciences, Little Rock, AR, USA; Knight Cardiovascular Institute, Oregon Health and Science University, Portland, OR, USA; Department of Medicine, Division of Cardiology, University of Arkansas for Medical Sciences, Little Rock, AR, USA; Department of Medicine, Division of Cardiology, University of Arkansas for Medical Sciences, Little Rock, AR, USA; Department of Cardiovascular Medicine, National Heart Centre, Singapore, Singapore; Knight Cardiovascular Institute, Oregon Health and Science University, Portland, OR, USA; Knight Cardiovascular Institute, Oregon Health and Science University, Portland, OR, USA; Department of Cardiology, Policlinico di Monza, Monza, Italy; Centro Cardiologico Monzino IRCCS, University of Milan, Milan, Italy; U.O.C. Risonanza Magnetica per Immagini, Fondazione G. Monasterio CNR-Regione Toscana Pisa, Pisa, Italy; U.O.C. Risonanza Magnetica per Immagini, Fondazione G. Monasterio CNR-Regione Toscana Pisa, Pisa, Italy; Department of Radiology, University Hospital Leuven, Leuven, Belgium; Cardiac Department, Vannini Hospital Rome, Rome, Italy; Department of Cardiology, Infermi Hospital, Rimini, Italy; Cardiovascular and Thoracic Department of Careggi Hospital, Florence, Italy; Department of Medical and Surgical Sciences, University of Foggia, Foggia, Italy; Maria Cecilia Hospital, GVM Care & Research, Cotignola, RA, Italy; Radiology Department, Parma University Hospital, Via Gramsci, Parma, Italy; Centro Cardiologico Monzino IRCCS, University of Milan, Milan, Italy; Division of Cardiothoracic Imaging, Emory University, Atlanta, GA, USA; Department of Cardiac, Thoracic, Vascular Sciences and Public Health University of Padua Medical School, Padova, Italy; Department of Cardiology, Policlinico di Monza, Monza, Italy; Radiology Department, Policlinico Casilino, Rome, Italy; Department of Radiology, University Hospital Leuven, Leuven, Belgium; Department of Medical Biotechnologies, Division of Cardiology, University of Siena, Siena, Italy; Department of Cardiology, Azienda Ospedaliero-Universitaria, Parma, Italy; Department of Medical Biotechnologies, Division of Cardiology, University of Siena, Siena, Italy; Department of Radiology, University of Foggia, Foggia, Italy; Centro Cardiologico Monzino IRCCS, University of Milan, Milan, Italy; Cardiology Department, Policlinico Casilino, Rome, Italy; Multimodality Cardiac Imaging Section, IRCCS Policlinico San Donato, San Donato Milanese, Milan, Italy; Institute of Management, Scuola Superiore Sant’Anna, Pisa, Italy; Department of Cardiology, Vall d’Hebron Institut de Recerca (VHIR), Universitat Auto`noma de Barcelona, Barcelona, Spain; Department of Cardiology, Policlinico di Monza, Monza, Italy; Scienze Radiologiche, Department of Medicine and Surgery, University of Parma, Parma, Italy; Department of Cardiology, Infermi Hospital, Rimini, Italy; School of Biomedical Engineering & Imaging Sciences, King’s College London, London, UK; De Gasperis’ Cardio Center, ASST Grande Ospedale Metropolitano Niguarda, Milan, Italy; Institute of Cardiovascular Disease, Department of Emergency and Organ Transplantation, University Hospital Policlinico of Bari, Bari, Italy; Department of Cardiology, ASST Monza, P.O. Desio, Italy; Centro Cardiologico Monzino IRCCS, University of Milan, Milan, Italy; Dipartimento Neuro-Cardiovascolare, Ospedale Ca’ Foncello Treviso, Treviso, Italy; Cardiovascular Department, CMR Center, University Hospital Lausanne, CHUV, Lausanne, Switzerland; Department of Radiology, Careggi Hospital, Florence, Italy; De Gasperis’ Cardio Center, ASST Grande Ospedale Metropolitano Niguarda, Milan, Italy; Centro Cardiologico Monzino IRCCS, University of Milan, Milan, Italy; Department of Cardiac, Thoracic, Vascular Sciences and Public Health University of Padua Medical School, Padova, Italy; Multimodality Cardiac Imaging Section, IRCCS Policlinico San Donato, San Donato Milanese, Milan, Italy; Division of Cardiology, Loyola University of Chicago, Chicago, IL, USA; Scienze Radiologiche, Department of Medicine and Surgery, University of Parma, Parma, Italy; Division of Cardiology, Loyola University of Chicago, Chicago, IL, USA; Department of Cardiology, Citta` della salute e della Scienza - Ospedale Molinette, Turin, Italy; Department of Cardiology, Vall d’Hebron Institut de Recerca (VHIR), Universitat Auto`noma de Barcelona, Barcelona, Spain; Radiology Department, Vannini Hospital Rome, Rome, Italy; Division of Cardiovascular Imaging, Department of Radiology and Radiological Science, Medical University of South Carolina, Charleston, SC, USA; Maria Cecilia Hospital, GVM Care & Research, Cotignola, RA, Italy; Scienze Radiologiche, Department of Medicine and Surgery, University of Parma, Parma, Italy; Department of Radiology, University Hospital Leuven, Leuven, Belgium; Division of Cardiology, Loyola University of Chicago, Chicago, IL, USA; UOC Radiologia, Ospedale “F. Spaziani”, Frosinone, Italy; U.O.C. Risonanza Magnetica per Immagini, Fondazione G. Monasterio CNR-Regione Toscana Pisa, Pisa, Italy; Department of Radiology, Fondazione IRCCS Policlinico S.Matteo, Pavia, Italy; Division of Cardiovascular Imaging, Department of Radiology and Radiological Science, Medical University of South Carolina, Charleston, SC, USA; Department of Cardiology, Citta` della salute e della Scienza - Ospedale Molinette, Turin, Italy; Centro Cardiologico Monzino IRCCS, University of Milan, Milan, Italy; Institute of Cardiovascular Disease, Department of Emergency and Organ Transplantation, University Hospital Policlinico of Bari, Bari, Italy; Cardiovascular Department, CMR Center, University Hospital Lausanne, CHUV, Lausanne, Switzerland; Faculty of Biology and Medicine, Lausanne University, UniL, Lausanne, Switzerland; Centro Cardiologico Monzino IRCCS, University of Milan, Milan, Italy

**Keywords:** heart failure, right ventricular dysfunction, cardiac magnetic resonance, heart failure hospitalization, ejection fraction

## Abstract

**Aims:**

Right ventricular systolic dysfunction (RVSD) is an important determinant of outcomes in heart failure (HF) cohorts. While the quantitative assessment of RV function is challenging using 2D-echocardiography, cardiac magnetic resonance (CMR) is the gold standard with its high spatial resolution and precise anatomical definition. We sought to investigate the prognostic value of CMR-derived RV systolic function in a large cohort of HF with reduced ejection fraction (HFrEF).

**Methods and results:**

Study cohort comprised of patients enrolled in the CarDiac MagnEtic Resonance for Primary Prevention Implantable CardioVerter DefibrillAtor ThErapy registry who had HFrEF and had simultaneous baseline CMR and echocardiography (*n* = 2449). RVSD was defined as RV ejection fraction (RVEF) <45%. Kaplan–Meier curves and cox regression were used to investigate the association between RVSD and all-cause mortality (ACM). Mean age was 59.8 ± 14.0 years, 42.0% were female, and mean left ventricular ejection fraction (LVEF) was 34.0 ± 10.8. Median follow-up was 959 days (interquartile range: 560–1590). RVSD was present in 936 (38.2%) and was an independent predictor of ACM (adjusted hazard ratio = 1.44; 95% CI [1.09–1.91]; *P* = 0.01). On subgroup analyses, the prognostic value of RVSD was more pronounced in NYHA I/II than in NYHA III/IV, in LVEF <35% than in LVEF ≥35%, and in patients with renal dysfunction when compared to those with normal renal function.

**Conclusion:**

RV systolic dysfunction is an independent predictor of ACM in HFrEF, with a more pronounced prognostic value in select subgroups, likely reflecting the importance of RVSD in the early stages of HF progression.

## Graphical Abstract

**Graphical Abstract:**
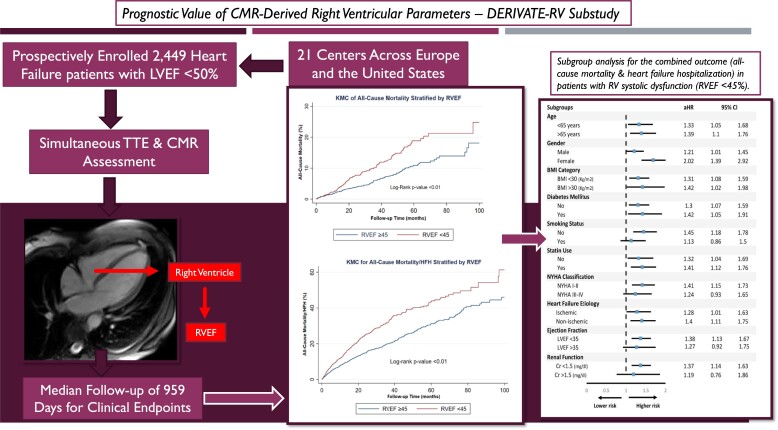

## Introduction

Heart failure (HF) is a heterogeneous disorder with a wide range of cardiomyopathies, which often cross the arbitrary left ventricular (LV) ejection fraction (EF) boundaries.^1^ The variable longitudinal trajectory of HF, coupled with the limited prognostic value of demographic and clinical data, necessitates the exploratory search for noninvasive imaging markers for better prognostication of incident adverse events, and for guidance of medical, percutaneous, and surgical therapies.

Right ventricular (RV) dysfunction has been recognized as an important determinant of clinical outcomes in HF cohorts.^2–4^ However, quantitative assessment of RV function is challenging in a routine clinical setting, as the geometrical complexity of the RV limits the ability of direct volumetric assessment by traditional two dimensional (2D) echocardiography. Other modalities have also been used for the evaluation of RV function, such as radionuclide ventriculography,^2^ right heart catheterization,^5^ and 3D echocardiography.^6^ Cardiac magnetic resonance (CMR), however, is the ‘gold standard’ for volumetric cardiac assessment and quantification due to its high level of spatial resolution, precise definition of anatomy, and excellent reproducibility.^7^ Few studies have investigated the prognostic value of CMR-derived RV volumetric parameters in HF with reduced EF (HFrEF).^2,8–12^ To this date, the significance of quantitative measures of RV dysfunction is not fully elucidated, primarily due to the small sample sizes and limited scope of the published data. Further, the incremental prognostic value of quantitative RV parameters of structure and function, on top of clinical parameters, is not known especially across various subgroups of HFrEF.

In this retrospective analysis, we utilized a large, multicentre, prospective cohort of HFrEF from the DERIVATE ‘CarDiac MagnEtic Resonance for Primary Prevention Implantable CardioVerter DefibrillAtor ThErapy’ registry.^13^ The primary objective was to explore the correlation between CMR-derived quantitative parameters of RV systolic function, mainly the RVEF, in predicting all-cause mortality (ACM) and HF hospitalizations (HFHs) amongst various subgroups of HFrEF patients.

## Methods

### DERIVATE registry

The design and rationale of the DERIVATE registry along with the protocols, inclusion and exclusion criteria are described in details in a previous publication.^13^ In brief, DERIVATE is an international, multicentre, prospective, observational study that enrolled consecutive HFrEF patients at 21 sites across Europe and the United States. Included patients underwent baseline evaluation with both transthoracic echocardiography (TTE) and CMR imaging.^13^ Inclusion criteria included the following: (i) age ≥18 years old, (ii) chronic HF with >3 months since the last decompensation, (iii) LVEF <50% at initial TTE evaluation, and (iv) both TTE and CMR are performed within 3 months of each other. Exclusion criteria included the following: (i) decompensated HF within 3 months of enrollment, (ii) recent myocardial infarction (<40 days), (iii) unstable angina, (iv) severe valvular disease, (v) hypertrophic cardiomyopathy, (vi) Takotsubo cardiomyopathy, (vii) cardiac amyloidosis, and (viii) congenital heart disease. The institutional ethical committees of the participating centres approved the protocol, and all patients gave written informed consent.

### Study design

The target population of DERIVATE was patients with clinical history of chronic HFrEF. Chronic HF was defined as >3 months from the last decompensated HF presentation according to the ACC/AHA classification.^14^ The ACC/AHA definition of HF with preserved LVEF had been established using a reference of LVEF ≥50%, and hence, this study included patients with HF and EF <50% (i.e. HFrEF). Severe LV dysfunction was defined as LVEF <35% according to the initial TTE evaluation. RV systolic dysfunction (RVSD) was defined as RVEF <45% by CMR based on cut-off used in previous publications.^8,10,12,15^ Image acquisition protocols for both TTE and CMR can also be found in previous publications.^13,16^

### Objectives and endpoints

The primary objective of the DERIVATE registry was to identify, quantify, and integrate CMR parameters with demographic, clinical, and TTE data for risk stratification in patients with HFrEF. The goal of present analysis was to investigate the correlation between CMR-derived quantitative parameters of RV systolic function, the RVEF, and clinical endpoints. ACM was the primary endpoint of the present analysis. The secondary endpoint was a composite outcome consisting of ACM and HFHs.

### Follow-up

Patient follow-up was performed at each local institution by dedicated personnel. The minimum follow-up period was 12 months. Quality control and study monitoring was performed in accordance with ICH-E6 Good Clinical Practice guidelines and applicable local regulations.

### Statistical analysis

The rationale for sample size determination of the DERIVATE registry was detailed in a prior publication.^13^ All statistical analyses were performed with the use of STATA 16 (State Corp LLC, College Station, Texas). A *p* value <0.05 was considered statistically significant. Baseline characteristics of patients were stratified according to RVSD (RVEF ≥45% vs. RVEF <45%). Descriptive statistics were used to characterize both groups. Student’s independent *t*-test, Chi-square, or Fischer’s exact test were used as appropriate to compare the distribution of continuous and categorical variables, respectively. Stratified according to RVSD, survival curves related to primary endpoints were plotted using the Kaplan–Meier (KM) method with right-censoring at 100 months due to a significant proportion of missing observations after that time period (57 of the 2449 study subjects had follow-up past 100 months). The log-rank test was used to assess for equality of survival functions.

Univariate Cox proportional hazard models were used to identify the variables associated with ACM. Significant variables (*P* value <0.05) at the univariate analyses were included in the final multivariable Cox proportional hazard models, in a stepwise fashion. The proportional-hazards assumption for Cox models was investigated based on Schoenfeld residual method as well as graphically. Results of the Cox proportional hazard models are reported as hazard ratios (HRs), and their correspondent 95% confidence intervals (CIs). Using the same covariates of the Cox models, subgroup analyses were conducted for study endpoints in patients with RVSD, and adjusted HHRs (aHRs) for various subgroups are summarized in forest plots.

Missing data for covariates were handled with the use of multiple imputation. Multiple imputation models incorporated all available baseline data. However, covariates with significant percentage (>20%) of missing data [i.e. tricuspid annular plane systolic excursion (TAPSE), pulmonary arterial systolic pressure (PASP), and pro-hormone B-type natriuretic peptide (Pro-BNP)] were not imputed or included in the Cox models. Rather, they were explored via KM curve subgrouping (Supplementary data online). The 10-fold cross validation method was used to assess the performance of the Cox proportional hazards regression model. The area under receiver operator curve (AUROC) was used as a performance measure of the model predictions and reported as the mean and standard deviation (SD) of the AUROC values.

## Results

A total of 2449 subjects with HFrEF were included in the analysis. Mean age was 59.8 ± 14.0 years and 42.0% were female. RVSD was present in 936 (38.2%) of the cohort. Mean LVEF was 34.0 ± 10.8 percent, 22.4% had a New York Heart Association (NYHA) class of III/IV, and 38.4% had ischaemic cardiomyopathy (ICM) as the underlying aetiology for the HF. Baseline characteristics are listed in *Table 1*. TTE and CMR studies were acquired in all patients with a median interval of 3 days [interquartile range (IQR): 2–5 days] between TTE and CMR. The median follow-up time for clinical endpoints was 959 days (IQR: 560–1590). TTE and CMR parameters of the study cohort, stratified by RVSD status, are summarized in *Table 2*.

**Table 1:** Baseline characteristics of the study cohort stratified by RVSD (defined as RVEF <45%)

	No RVSD (*N* = 1513)	RVSD (*N* = 936)	Total Cohort (*N* = 2449)	*P*-value
**General characteristics**				
ȃAge (mean ± SD, years)	59.4 ± 13.9	60.5 ± 14.0	59.8 ± 14.0	0.07
ȃAge > 65 years, *n***(%)**	622 **(41.1)**	407 **(43.5)**	1029 **(42.0)**	0.09
ȃFemale; *n***(%)**	418 **(27.6)**	168 **(17.9)**	586 **(23.9)**	<0.01
ȃBMI (mean ± SD, Kg/m^2^)	26.4 ± 4.5	26.8 ± 4.9	26.5 ± 4.6	0.07
ȃBSA (mean ± SD, m^2^)	1.88 ± 0.27	1.91 ± 0.24	1.89 ± 0.27	0.01
ȃFamily history of CAD; *n***(%)**	448/1472 **(30.4)**	266/823 **(32.3)**	714/2295 **(31.1)**	0.55
ȃSmoker; *n***(%)**	556 **(36.8)**	409 **(43.7)**	965 **(39.4)**	<0.01
ȃHypertension; *n***(%)**	763 **(50.4)**	464 **(49.6)**	1227 **(50.1)**	0.68
ȃHyperlipidemia; *n***(%)**	649/1482 **(43.8)**	348**/**824 **(42.2)**	997**/**2306 **(43.2)**	0.24
ȃDiabetes Mellitus; *n***(%)**	285 **(18.8)**	245 **(26.2)**	530 **(21.6)**	<0.01
ȃCreatinine (mean ± SD, mg/dL)	1.02 ± 0.34	1.12 ± 0.43	1.1 ± 0.4	<0.01
ȃLeft bundle branch block	371 **(24.5)**	242 **(25.9)**	613 **(25.0)**	0.44
**Symptom burden (NYHA class)**				
ȃNYHA class I/II; *n***(%)**	1246 **(82.4)**	654 **(69.8)**	1900 **(77.6)**	<0.01
ȃNYHA class III/IV; *n***(%)**	267 **(17.6)**	282 **(30.1)**	549 **(22.4)**	<0.01
**Aetiology of** HF				
ȃICM; *n***(%)**	465 **(30.7)**	475 **(50.8)**	940 **(38.4)**	<0.01
ȃIdiopathic/dilated CM; *n***(%)**	1028 **(69.3)**	461 **(49.2)**	1509 **(61.6)**	<0.01
**Medications**				
ȃDiuretics; *n***(%)**	1028 **(68.0)**	681 **(72.7)**	1709 **(69.7)**	0.02
ȃStatin; *n***(%)**	684 **(45.2)**	504 **(53.8)**	1188 **(48.5)**	<0.01
ȃAnti-platelet; *n***(%)**	811 **(53.6)**	502 **(53.6)**	1313 **(53.6)**	0.99
ȃAnti-coagulation; *n***(%)**	310 **(20.5)**	213 **(22.8)**	523 **(21.4)**	0.20
ȃACE-I/ARB; *n***(%)**	1230 **(81.3)**	854 **(91.2)**	2084 **(85.1)**	<0.01
ȃBeta blocker; *n***(%)**	1250 **(82.6)**	834 **(89.1)**	2084 **(85.1)**	<0.01
ȃAny antiarrhythmic agent; *n***(%)**	315 **(20.8)**	115 **(12.2)**	430 **(17.6)**	<0.01

**Table 2:** TTE and cardiac MRI (CMR) parameters of the 2449 patients with HF stratified by RVSD (RVEF <45% vs. RVEF ≥45%)

	No RVSD (*N* = 1513)	RVSD (*N* = 936)	Total Cohort (*N* = 2449)	*P*-value
**TTE parameters**				
ȃLVEF (mean ± SD, %)	36.5 ± 10.1	30.0 ± 10.6	34.0 ± 10.8	<0.01
ȃLVEDV/BSA (mean ± SD, mL/m^2^)	94.7 ± 34.1	104.4 ± 38.5	98.1 ± 36.0	<0.01
ȃLVESV/BSA (mean ± SD, mL/m^2^)	61.2 ± 27.8	74.7 ± 33.3	66.0 ± 30.6	<0.01
ȃTAPSE (mean ± SD, mm)	20.8 ± 4.2	17.8 ± 4.8	19.8 ± 4.7	<0.01
ȃPASP (mean ± SD, mmHg)	32.3 ± 10.5	39.1 ± 13.7	34.9 ± 12.3	<0.01
ȃDiastolic dysfunction; *n* (%)	256/1199 (21.4)	210/571 (36.8)	466/1770 (26.3)	<0.01
**CMR parameters**				
ȃCMR-LVEF (mean, %)	35.5 ± 10.1	25.3 ± 9.9	31.6 ± 11.2	<0.01
ȃCMR-LVEDV/BSA (mL/m^2^)	123.4 ± 37.7	136.3 ± 46.7	128.3 ± 41.8	<0.01
ȃCMR-LVESV/BSA (mL/m^2^)	81.4 ± 34.1	103.8 ± 43.1	90.0 ± 39.3	<0.01
ȃCMR-LVSV (mean, mL)	79.3 ± 26.5	62.2 ± 24.1	72.8 ± 26.9	<0.01
ȃCMR-LV mass/BSA (g/m^2^)	78.9 ± 25.9	80.2 ± 28.6	79.4 ± 26.9	0.29
ȃCMR-RVEDV/BSA (mL/m^2^)	69.2 ± 20.6	86.2 ± 39.2	75.0 ± 29.4	<0.01
ȃCMR-RVESV/BSA (mL/m^2^)	29.7 ± 11.7	56.9 ± 29.7	40.1 ± 24.4	<0.01
ȃCMR-RVEF (mean, %)	57.9 ± 7.8	33.2 ± 8.9	48.4 ± 14.5	<0.01
ȃCMR-RVSV (mean, mL)	74.9 ± 23.5	53.0 ± 24.9	66.5 ± 26.3	<0.01

### Association between RVEF and clinical endpoints

**Figure 1:**
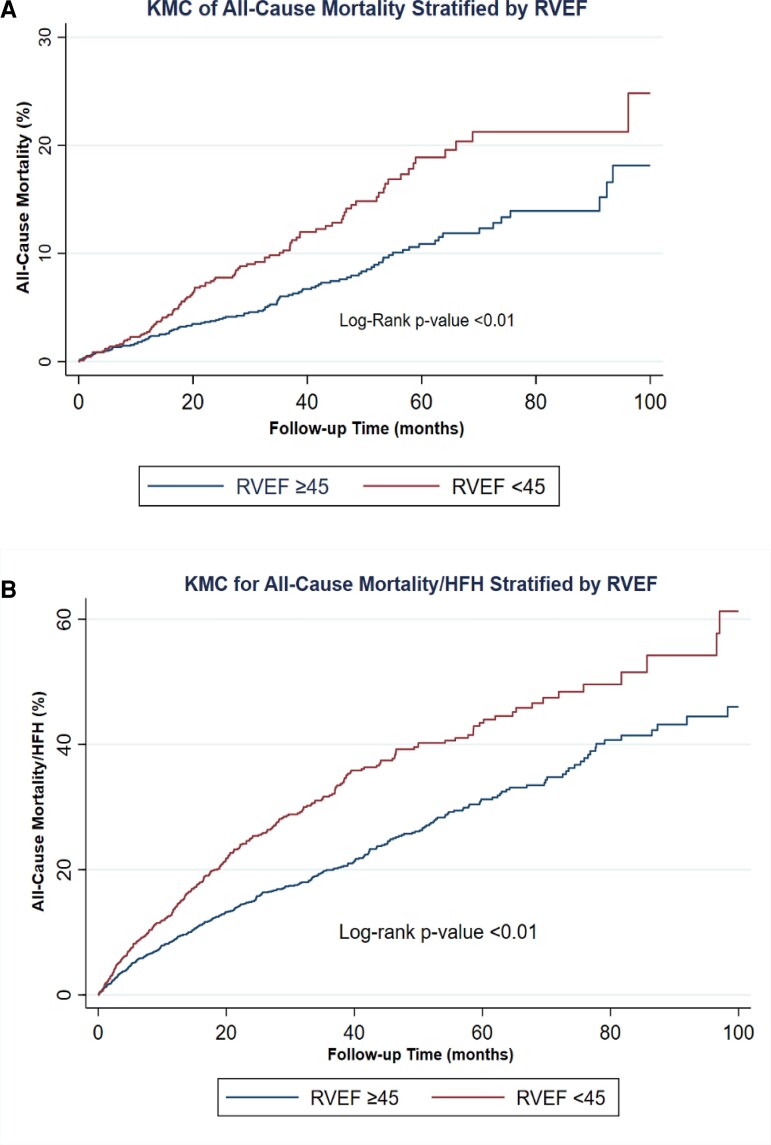
KM curve analysis for the primary outcome of ACM (*A*) and the composite secondary outcome of ACM and/or HFH (*B*), stratified by RVEF.

**Figure 2:**
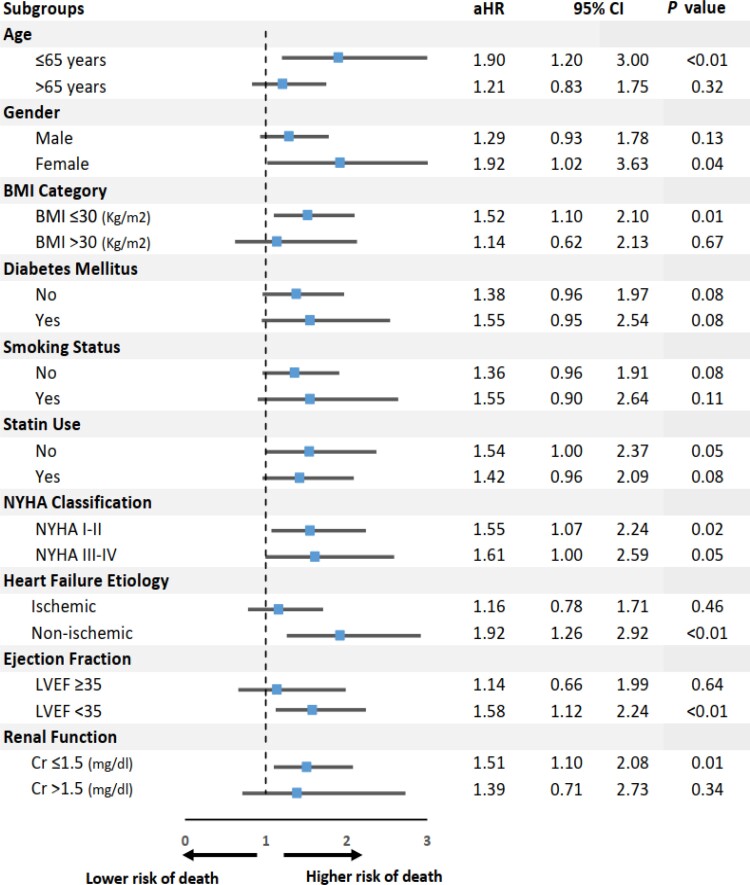
Subgroup analysis with aHRs for the primary outcome of ACM in patients with RVSD (defined as EF <45%).

At 100 months of follow-up, ACM occurred in 212 (8.7%) patients, of which 104 patients had RVSD and 108 patients had normal RVEF (non-RVSD). Mortality rate was significantly higher in patients with RVSD (104/936; 11.1%) compared to non-RVSD patients (108/1513, 7.1%); *P* < 0.01. This is also shown in KM curves (*Figure 1A*). RVSD was associated with higher ACM with aHR of 1.44 (95% CI; 1.09–1.91; *P* = 0.01) in the multivariable analysis (*Table 3*). Advanced age (>65 years), diabetes mellitus, smoking status, renal impairment (creatinine >1.5 mg/dL), NYHA class III/IV, and ICM were independently associated with significantly higher ACM (*Table 3*). Results of subgroup analysis for the primary outcome of ACM are shown in *Figure 2*.

**Table 3:** Univariate (model 1) and multivariate (model 2) cox-regression analysis in the study cohort for the primary outcome of ACM and the composite secondary outcome of ACM and HFH

Variables	ACM (univariate analysis)		ACM (multivariate analysis)		Composite outcome (univariate analysis)		Composite outcome (multivariate analysis)	
	HR [95% CI]	*P* value	HR [95% CI]	*P* value	HR [95% CI]	*P* value	HR [95% CI]	*P* value
RVEF <45	**1.74 [1.33–2.28]**	<0.01	1.44 [1.09–1.91]	0.01	1.61 [1.38–1.88]	<0.01	1.40 [1.19–1.64]	<0.01
Age >65	**2.17 [1.64–2.85]**	<0.01	1.58 [1.17–2.15]	<0.01	1.44[1.23–1.68]	<0.01	1.23 [1.04–1.46]	0.02
Female gender	**0.94 [0.68**–**1.29]**	0.70	1.03 [0.74–1.44]	0.85	0.81[0.67–0.97]	0.03	0.86 [0.71–1.05]	0.13
BMI ≥30	**1.04 [0.74**–**1.45]**	0.83	0.90 [0.64–1.28]	0.56	1.38[1.15–1.65]	<0.01	1.25 [1.03–1.50]	0.02
Hypertension	**1.42 [1.08**–**1.87]**	0.01	1.01 [0.75–1.36]	0.98	1.18[1.01–1.37]	0.04	0.94 [0.79–1.11]	0.44
Diabetes mellitus	**1.97 [1.49**–**2.62]**	<0.01	1.47 [1.08–1.99]	0.01	1.74[1.47–2.07]	<0.01	1.41 [1.19–1.71]	<0.01
Smoking status	**0.69 [0.51**–**0.92]**	0.01	0.64 [0.47–0.87]	<0.01	0.83[0.71–0.98]	0.03	0.77 [0.62–0.91]	<0.01
NYHA III/IV	**2.39 [1.81**–**3.16]**	<0.01	2.10 [1.58–2.79]	<0.01	2.01[1.71–2.37]	<0.01	1.81 [1.53–2.15]	<0.01
Creatinine >1.5	**2.59 [1.85**–**3.63]**	<0.01	1.74 [1.22–2.48]	<0.01	1.90[1.52–2.37]	<0.01	1.41 [1.12–1.77]	<0.01
Ischaemic CM	**1.82 [1.39**–**2.30]**	<0.01	1.39 [1.03–1.87]	0.03	1.40[1.20–1.64]	<0.01	1.13 [0.95–1.34]	0.09

**Model 1:** Unadjusted – RVSD-status only model. **Model 2:** Adjusted for age, gender, BMI, hypertension, diabetes mellitus, kidney function, NYHA Class, HF aetiology (ischaemic vs. non-ischaemic), and smoking status.

**Figure 3:**
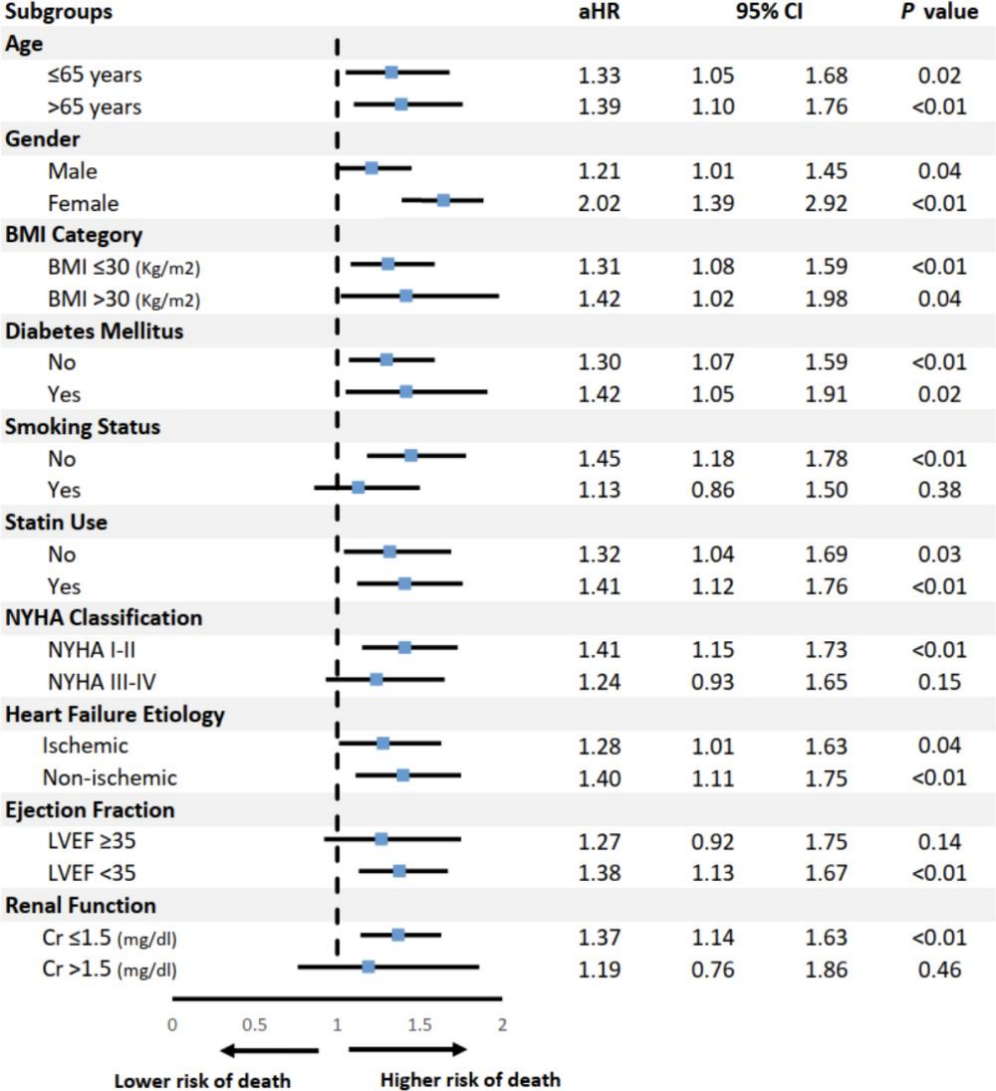
Subgroup analysis with aHRs for the composite outcome of ACM and/or HFHs in patients with RVSD (defined as EF <45%).

The composite outcome of ACM and/or HFH occurred in 645 (35.8%) patients at 100 months of follow up and was more prevalent in patients with RVSD compared to non-RVSD (31.9% vs. 22.9%, *P* value <0.01). KM curves are shown in *Figure 1B*. RVSD was associated higher ACM and/or HFH, with an aHR of 1.40 (95% CI; 1.19–1.64; *P* < 0.01) in the multivariable analysis (*Table 3*). Advanced age (>65 years), higher body mass index (BMI ≥30), diabetes mellitus, smoking status, renal impairment, and NYHA class III/IV were independent predictors of the composite outcome (*Table 3*). Results of subgroup analysis for the composite outcome are shown in *Figure 3*.

Results from 10-fold cross-validation analysis are shown in supplementary figures (see Supplementary data online, *Figure S5 a and b*). The mean cross-validation AUROC for the ACM model was 0.67 (95% CI: 0.61–0.70), with a SD of 0.08. The mean cross validations AUROC for the composite outcome model was 0.64 (95% CI: 0.61–0.66), with a SD of 0.04.

### Subgroup analysis for the association between RVEF and clinical endpoints

#### Effect of LVEF on outcomes stratified by RV systolic function

Supplementary data online, *Figure S1* depicts the linear correlation between RVEF and LVEF (*r* = 0.29, *P* < 0.01). Severe LV systolic dysfunction (LVSD) was independently associated with increased risk of ACM and/or HFH (HR = 1.53, 95% CI: 1.29–1.81, *P* < 0.01). In subgroup analysis based on LVSD severity, RVSD was found to be independently predictive of ACM (HR = 1.58, 95% CI: 1.12–2.24, *P* < 0.01) and the composite outcome of ACM and/or HFH (HR = 1.38, 95% CI 1.13–1.67, *P* < 0.01) only in the severe LVSD group (LVEF <35%). However, it did not reach statistical significance for ACM or the composite outcome in patients with LVEF ≥35%, *Figures 2 and 3*. KM curves for ACM in RVSD groups stratified by LVEF are shown in supplementary*Figure S3* (see Supplementary data online, *Figure S3g*).

#### Effect of HF aetiology on outcomes stratified by RV systolic function

In the present cohort, non-ICM (NICM) was present in 61.6% of patients (*Table 1)*. Compared to NICM group, ICM was independently associated with an increased risk of ACM with an aHR of 1.40 (95% CI: 1.20–1.64, *P* < 0.02) in the multivariable model (*Table 3*). In subgroup analysis based on HF aetiology, RVSD was predictive of ACM in patients with NICM (aHR = 1.92, 95% CI: 1.26–2.92, *P* < 0.01), but not in patients with ICM (aHR = 1.16, 95% CI: 0.78–0.71, *P = 0.46*), *Figure 2*. For the secondary outcome, RVSD was predictive of ACM and/or HFH in both ICM and NICM subgroups (*Figure 3*). KM curves for ACM in RVSD groups stratified by HF aetiology are shown in supplementary*Figure S3* (see Supplementary data online, *Figure S3b*).

#### Effect of NYHA class on outcomes stratified by RV systolic function

Advanced NYHA class (III/IV) was more prevalent in patients with RVSD compared to those with normal RV systolic function (30.1% vs. 17.6%) (*Table 1*). Advanced NYHA class (III/IV) was independently associated with increased risk of ACM (aHR = 2.10, 95% CI: 1.58–2.79, *P* < 0.01) and the composite outcome of ACM and/or HFH (aHR = 1.81, 95% CI: 1.53–2.15, *P* < 0.01) (*Table 3*). Upon subgroup analysis based on NYHA class, RVSD was predictive of ACM irrespective of NYHA class category (*Figure 2*). However, for the composite outcome, RVSD was associated with worse outcomes in NYHA I/II group (aHR = 1.41, 95% CI: 1.15–1.73, *P* < 0.01), but not in NYHA III/IV group (aHR = 1.24, 95% CI: 0.93–1.65, *P* = 0.15), *Figure 3*. KM curves for ACM in RVSD groups stratified by NYHA class are shown in supplementary*Figure S3* (see Supplementary data online, *Figure S3a*).

## Discussion

The prognostic role of RV dysfunction in HF is well established, however, the significance of this relationship in specific subgroups and phenotypes of HF patients has not been well-validated. Hereby, we present our analysis that uses a large multicentre prospective cohort to comprehensively explore the prognostic role of CMR-derived RV systolic function in different subgroups of a HFrEF cohort. Our results demonstrate that RVSD (defined as RVEF ≤45% by CMR) is prevalent amongst chronic HF patients (38.2%) and is an independent predictor of ACM and the composite outcome of HFH/ACM, even after adjusting for LV dysfunction and multiple other covariates. Several studies have evaluated the prognostic role of RVEF in HF patients using different modalities and cut-offs to define RVSD (*Table 4*).^2,4,6,8–12,15,17–19^

**Table 4:** Summary of major studies investigating the prognostic value of RVEF

Study/year	RVSD definition cut-off-modality	population	*n*	follow-up	Main finding
Larose *et al*. 2007^4^	<40% (CMR)	Patients with recent myocardial infarction (>30 days)	147	Median 17 months	RVEF <40% remained a significant independent predictor of mortality after adjusting for LVEF and infarct size (aHR 2.86; *P* = 0.03)
Meyer *et al*. 2010^2^	Multiple cut-offs: <40, <30, <20 (Radionuclide ventriculography)	Chronic HFrEF patients from ‘BEST’ trial	2008	Mean 2 years	RVSD was independently associated with mortality only at the cut-off <20%, aHR 1.32 (1.02 to 1.71; *P* = 0.034)
Gulati *et al*. 2013^8^	<45% (CMR)	Chronic HFrEF patients with dilated NICM	250	Median 6.8 years	RVSD was an independent predictor of mortality or cardiac transplant (HR 3.90; 95% CI: 2.16–7.04; *P* < 0.01)
Murninkas *et al*. 2014^9^	<38% (Radionuclide angiography)	Stable outpatient HFrEF cohort	246	Median 2.7 years	RVSD was not significantly associated with MACE or death after adjusting for LVEF and age
Goliasch *et al*. 2015^17^	<35% (CMR)	Chronic HFpEF patients	142	Median 10 months	RVSD was associated with hospitalization and cardiac death on univariate analysis, but not after adjusting for covariates.
Aschauer *et al*. 2016^15^	≤45% (CMR)	Chronic HFpEF patients	171	Median 1.5 years	RVSD was an independent predictor of MACE (HR 4.90; 95% CI: 2.46–9.75; *P* < 0.01)
Mikami *et al*. 2017^10^	<45% (CMR)	Chronic HFrEF patients (ischaemic and non-ischaemic)	314	Median 2.1 years	RVSD was an independent predictor of cardiac arrest and/or ICD implantation (HR = 2.98; *P* = 0.002)
Gill *et al*. 2019^11^	<20% (CMR)	HFrEF patients with LVEF ≤35	87	Median 3 years	RVSD was associated with a higher risk of MACE in the NICM subgroup but not ICM subgroup.
Purmah *et al*. 2021^18^	<40% (CMR)	Broad cardiovascular disease population, mean LVEF 55%	7131	Median 2.48 years	RVSD was associated with unadjusted HR of 3.1 for MACE, however it was not statistically significant after adjusting for LVEF
Ashcroft *et al*. 2021^6^	<46.9% (3D echocardiography)	Patients admitted with acute HF	418	Median 2 years	RVSD was associated with increased risk of ACM (HR 1.48; 95% CI 1.09–2.03, *P* ≤ 0.01)
Becker *et al*. 2021^12^	<45% (CMR)	Stable patients with dilated cardiomyopathy, mean LVEF 37% [25–44%]	216	Median 2.2 years	RVSD was significantly associated with shorter time to the composite of death and ventricular arrhythmias (10% drop in RVEF was associated with 0.81 increase in aHR, *P* = 0.02)
Kanagala *et al*. 2021^19^	<47% (CMR)	Chronic HFpEF patients compared against healthy controls	183	Median 4 years	RVSD was a strong independent predictor of HFH/ACM (aHR = 3.95, 95% CI: 1.88–8.29, *P* < 0.001)

Gulati *et al*.^8^ (*n* = 250 patients, median follow-up 6.8 years) investigated patients with dilated NICM and reduced LVEF <50%. An RVEF ≤45% was found to be an independent predictor of mortality or cardiac transplant in this group of patients (HR 3.90; 95% CI: 2.16–7.04; *P*-value <0.01).

Purmah *et al*.^18^ investigated the prognostic significance of RVEF in a broad non-specific cardiovascular population. An RVEF <40% was associated with an unadjusted HR of 3.1 for composite outcome of major cardiovascular events but was not statistically significant after adjustment for LVEF. Other studies have also evaluated the role of RVEF in HF patients with preserved EF (HFpEF).^15,17,19^

### Defining RV dysfunction

Multiple echocardiographic parameters have been extensively studied and validated as surrogates of RV function, such as TAPSE, right ventricular systolic excursion velocity (RV S’), fractional area change (FAC), RV index of myocardial performance, RV longitudinal peak systolic strain, and semi-quantitative RV function.^20^ However, there is no single parameter that is universally acceptable to define RV dysfunction, since some can be operator dependent with limited reproducibility. In addition, the established geometrical differences in contractile function between RV and LV with the general assumption that RV function is mainly longitudinal rather than circumferential has driven the development of such parameters. However, assessing RV longitudinal function alone might not be enough to prognosticate HF patients, especially in the setting of load altering therapies or in the setting of significant LV dysfunction, where remodelling might alter the way in which the RV functions.^21^ The significance of RV dysfunction in stable HF patients remains not well-understood despite being commonly diagnosed by these methods. In this study, we chose the cut-off of 45% for CMR-RVEF to define RV dysfunction based on some published studies.^8,10,12,15^ Other studies have used different cut-offs to define RV dysfunction as summarized in (*Table 4*).

### Relationship between RVEF and LVEF

The present study demonstrated a positive correlation between RVEF and LVEF in this HFrEF cohort (*r* = 0.29, *P* < 0.001) (Supplementary data online, *Figure S1*). Patients were stratified into severe and non-severe LVSD using a cut-off LVEF of 35% by echocardiography. RVSD was generally associated with worse outcomes in both groups; however, it reached statistical significance only in the severe LVSD group. This might be attributed to multiple factors. First, the DERIVATE cohort is different from the general HF population, in the sense that this study was mainly selecting patients with stable chronic HF while excluding patients with normal LVEF and those with severe valvular disease. In addition, the measurement of LVEF in this population might have been more accurate than the general population, as a result of the use of CMR, resulting in different accuracy for classifying patients into severe LVSD and non-severe LVSD, since there was no blinding for echocardiography interpreters from CMR data. More importantly, the loss of longitudinal contractile function of RV in severe LV dysfunction—as discussed in the previous section - can make RV function mainly dependent on circumferential contraction, which is better assessed by RVEF.

Finally, it is important to consider that while it is commonly established that right-sided HF could be a late manifestation of left-sided HF. However, due to interventricular dependence and activation of the neurohormonal system, RV dysfunction can also lead to LV dysfunction, ranging from relaxation abnormalities to LV systolic failure and electrophysiologic remodelling.^22^

### RV dysfunction and pulmonary hypertension

The ratio of maximum ventricular elastance (Ees) to arterial elastance (Ea) is an established measure of RV-PA coupling, which describes the efficient transfer of potential energy from one elastic chamber (RV) to another (PA). These values are typically derived from right heart catheterization pressure-volume loops. The ideal Ees/Ea ratio ranges between 1 and 2, and a drop in this ratio below 0.8 is suggestive of ‘uncoupling’ and RV maladaptation.^23^ CMR can offer a non-invasive way of estimating this ratio by the so-called ‘volume method’, wherein the ratio of stroke volume (SV) to end systolic volume (ESV) has been shown to correlate well with Ees/Ea ratio. Tello *et al*.^24^ proposed that a drop in SV/ESV below 0.805 can predict RV dysfunction (defined as RVEF <35%) with a sensitivity of 65.4% of and specificity of 87.5%. This is an expected correlation since both variables are partial products of the inert contractility of the RV. Our data redemonstrated the strong correlation between CMR-derived coupling variable (RVSV/ESV ratio) and RVEF (R = 0.91; *P* < 0.0001) (see Supplementary data online, *Figure S4*).

In addition, the prognostic significance of RVSD after adjusting for PASP in HF patients has been questionable.^25,26^ In this analysis, we could not adjust for PASP due to high missingness rate. RVSD was significantly associated with higher mortality in in patients with elevated PASP (>35 mmHg), but not in those with normal PASP (PASP ≤35 mmHg) on survival analysis of subgroups (see Supplementary data online, *Figure S3c*). This could be due to the co-existence of pulmonary hypertension with advanced HF.^27^ For instance, the prevalence of advanced (symptomatic) HF (NYHA III/IV) in the present cohort was 32.7% in those with elevated PASP compared to 17.1% in those with normal PASP.

### Limitations

First, the patient population was restricted to HFrEF patients, which precludes the ability to make conclusions on RVSD in HFpEF population. Second, we could not adjust for surrogates of pulmonary hypertension, such as PASP or TAPSE, in the Cox regression models due to the high missingness rates as they were not routinely reported on echocardiography. Alternatively, we performed subgroups analysis stratifying the cohort by PASP (elevated PASP defined as >35 mmHg) and TAPSE (abnormal TAPSE defined as <17 mm). Third, our study excluded patients with decompensated HF (NYHA class IV) within the past 3 months, as well as patients with recent myocardial infarction (<40 days) and unstable angina. This would likely have introduced selection bias in the subgroup analyses that partially explains why RVSD was not predictive of mortality in NYHA III/IV and ICM subgroups. Fourth, the observational nature of our data precludes the ability to make conclusion on causal association of RV dysfunction with clinical outcomes. In addition, external validation using a separate dataset is still required to verify the prognostic significance of RV parameters in HF patients. Finally, subgroup analysis is not a commonly adopted approach with observational data, however, epidemiologic studies suggest that subgroup-specific effects based on observational data could still be comparable to those performed in randomized clinical trials.^28^

## Conclusions

In patients with HFrEF, RV dysfunction is an independent predictor of poor clinical outcomes (HFH/ACM), irrespective of HF aetiology (ICM versus NICM). CMR-derived quantitative assessment of RV function can provide valuable prognostic information and improve risk stratification of HF patients. However, the prognostic value of RVSD appears to have subgroup-specific effects; for instance, it was more pronounced in patients with NYHA I/II as opposed to those with NYHA III/IV, in patients with LVEF <35% as opposed to those with LVEF ≥35%, and in patients with normal renal function as opposed to those with renal dysfunction. These findings could reflect the importance of RV function in the early stages of HF, prior to the onset of clinical and hemodynamic deterioration.

## Supplementary Material

jeac124_Supplementary_Data

## Data Availability

The data underlying this article will be shared on reasonable request to the corresponding author.
