# Supplementary material for: Cardiac magnetic resonance for prophylactic implantable-cardioverter defibrillator therapy international study: prognostic value of cardiac magnetic resonance-derived right ventricular parameters substudy
Source: Eur Heart J Cardiovasc Imaging. 2022 Jul 6;24(4):472–82. doi: 10.1093/ehjci/jeac124 (PMC10029842; doi:10.1093/ehjci/jeac124)
Supplement: jeac124_Supplementary_Data [file jeac124_supplementary_data.docx]

**SUPPLEMENTARY DATA**

Figure S1: Correlation between right ventricular ejection fraction (RVEF) and left ventricular ejection fraction (LVEF).

**Figure S2:** Correlation between RVEF and pulmonary artery systolic pressure (PASP).

**Figure S3:** Kaplan Meier Curve (KMC) of all-cause mortality in patients with RVSD (RVEF <45%) vs. normal RV systolic function (RVEF ≥45%) across the following subgroups:

1. NYHA Class (class I-II vs. III-IV)
2. Etiology (ischemic vs. non-ischemic)
3. Pulmonary artery systolic pressure (PASP <35 vs. PASP ≥35 mmHg)
4. Tricuspid Annular Plane Systolic Excursion (TAPSE <17 mm vs. TAPSE ≥17 mm)
5. Presence of diabetes mellitus
6. Presence of kidney injury (creatinine ≥1.5 mg/dL vs. <1.5 mg/dL).
7. Left ventricular ejection fraction (LVEF) (LVEF <35 vs. LVEF ≥35)

**Figure S4:** Correlation between right ventricular ejection fraction (RVEF) and the ratio of right ventricular systolic volume (RVSV) and right ventricular end-systolic volume (RVESV).

**Figure S5:** 10-fold cross validation method for regression model for:

1. All-cause mortality
2. Composite outcome of all-cause mortality/HFH

**Figure S1:** Correlation between right ventricular ejection fraction (RVEF) and left ventricular ejection fraction (LVEF). The continuous line represents the fitted linear regression line.


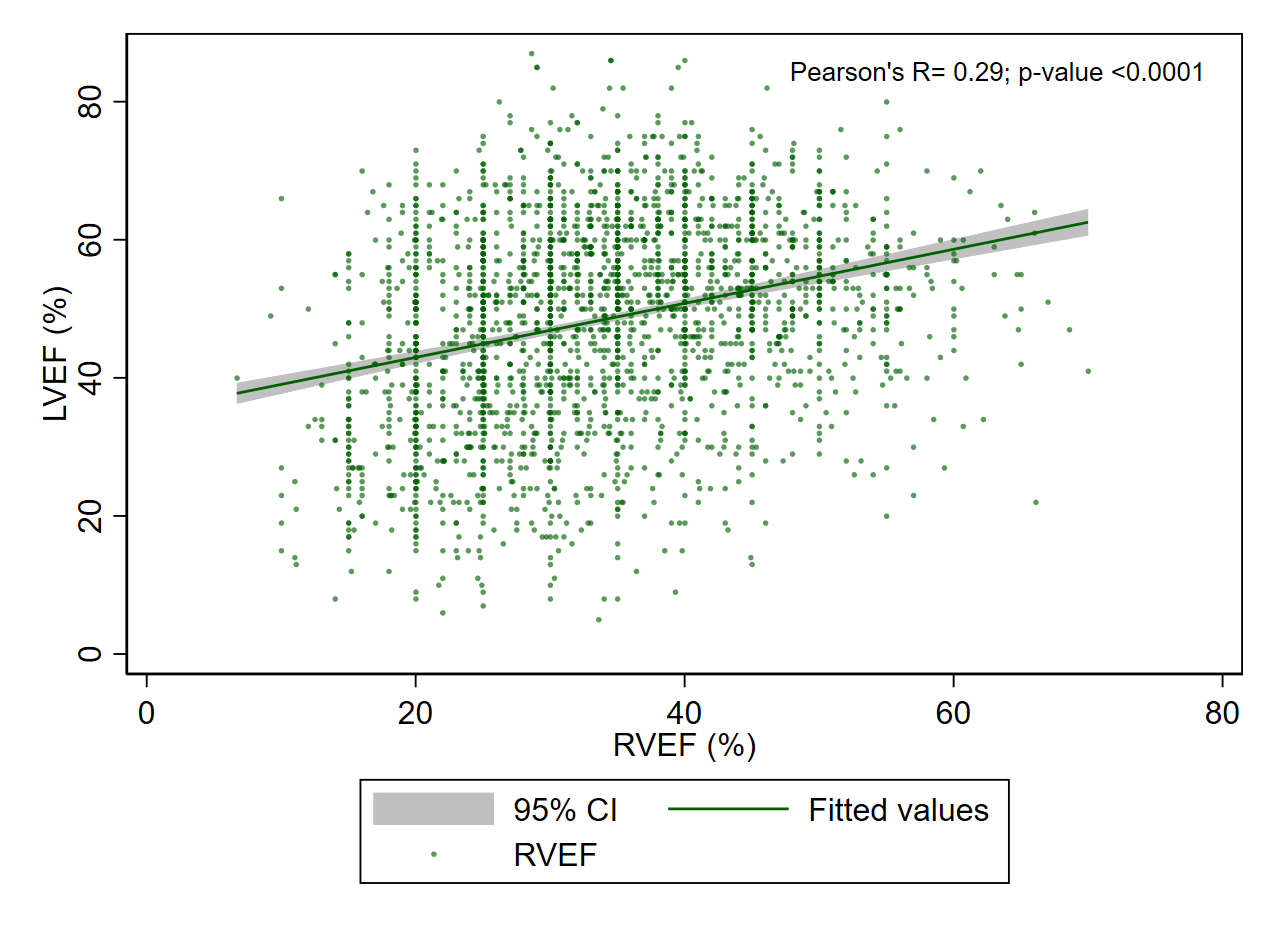


**Figure S2:** Correlation between right ventricular ejection fraction (RVEF) and pulmonary artery systolic pressure (PASP). The continuous line represents the fitted linear regression line.


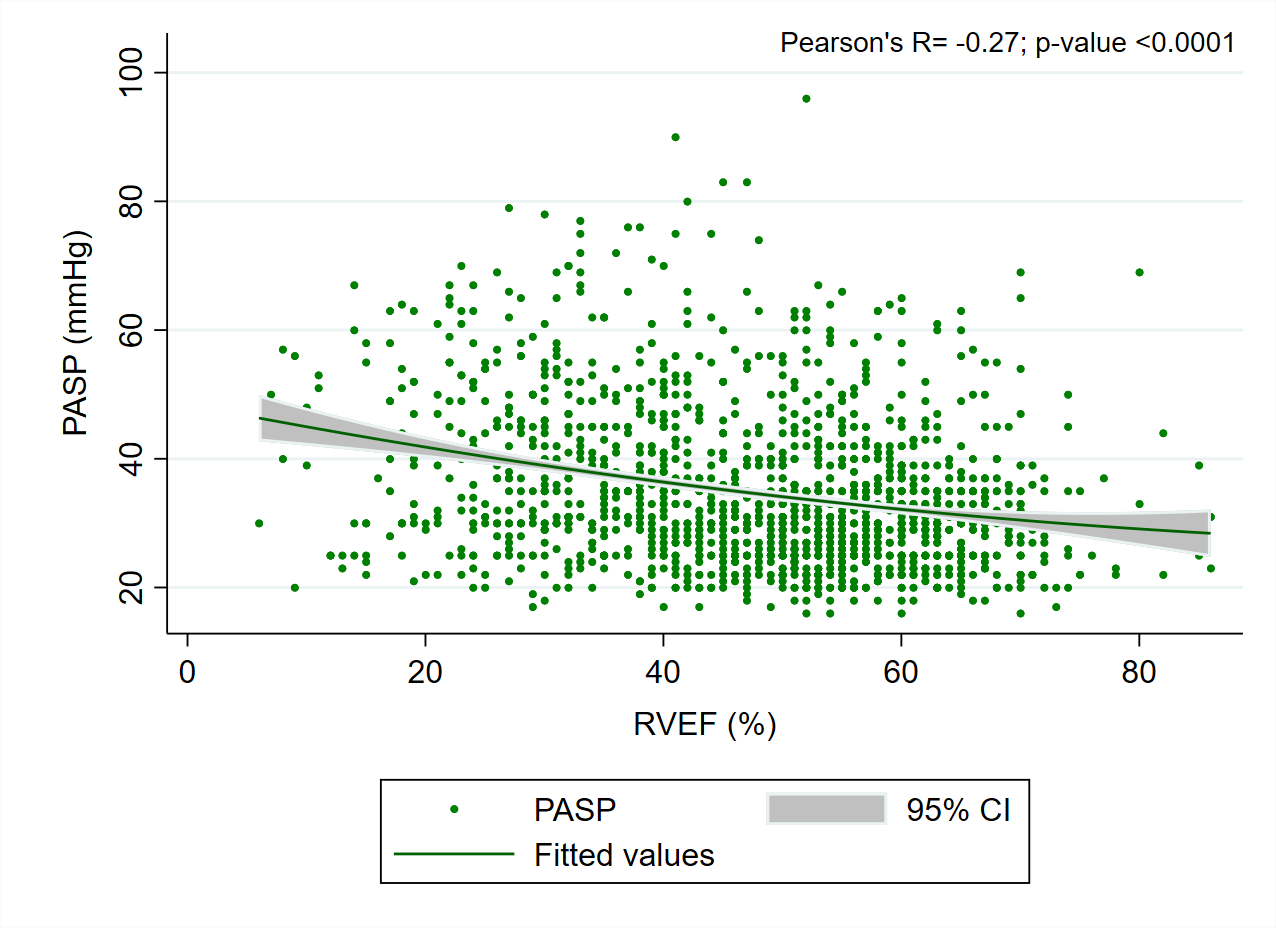


**Figure S3:** Kaplan Meier Curve (KMC) of all-cause mortality in patient with RVSD (RVEF <45%) vs. normal RV function (RVEF ≥45%) across the following subgroups: (a) NYHA Class, (b) etiology (ischemic vs. non-ischemic), (c) pulmonary artery systolic pressure (PASP <35 vs. PASP ≥35 mmHg), (d) Tricuspid Annular Plane Systolic Excursion (TAPSE <17 mm vs. TAPSE ≥17 mm), (e) presence of diabetes mellitus, (f) presence of kidney injury (creatinine <1.5 mg/dL vs. ≥1.5 mg/dL), and (g) LVEF <35%.

**Figure S3-a: NYHA Class**

**
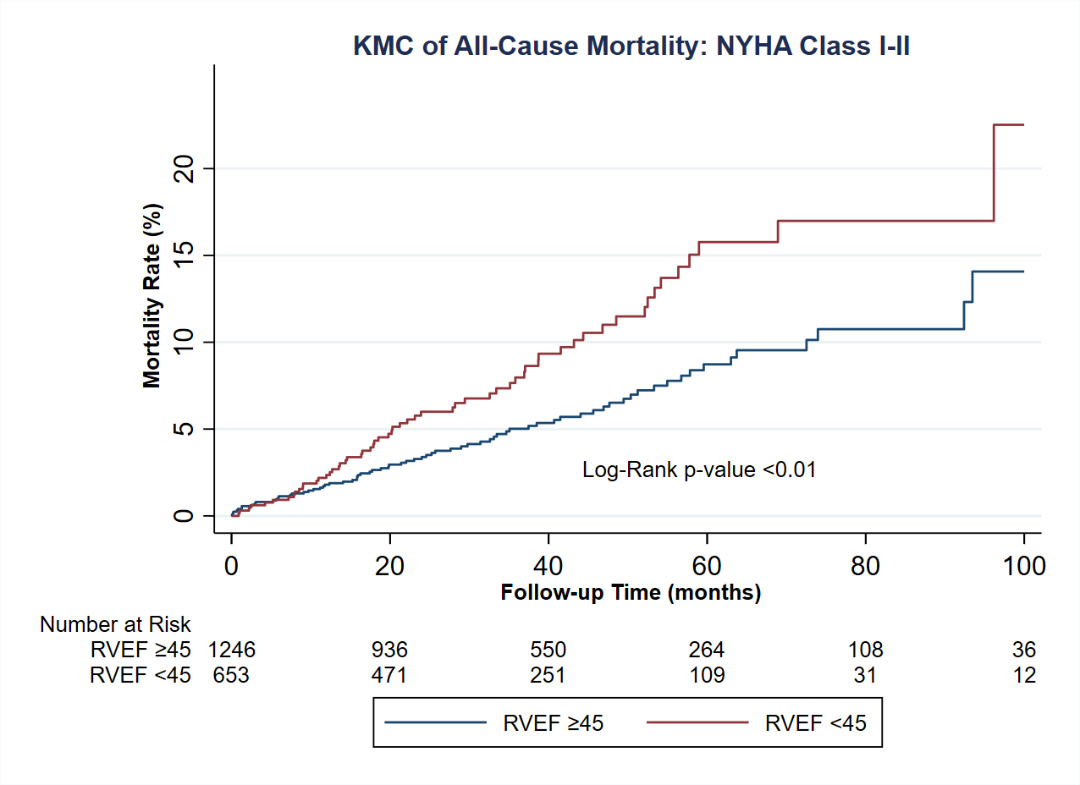
**

**
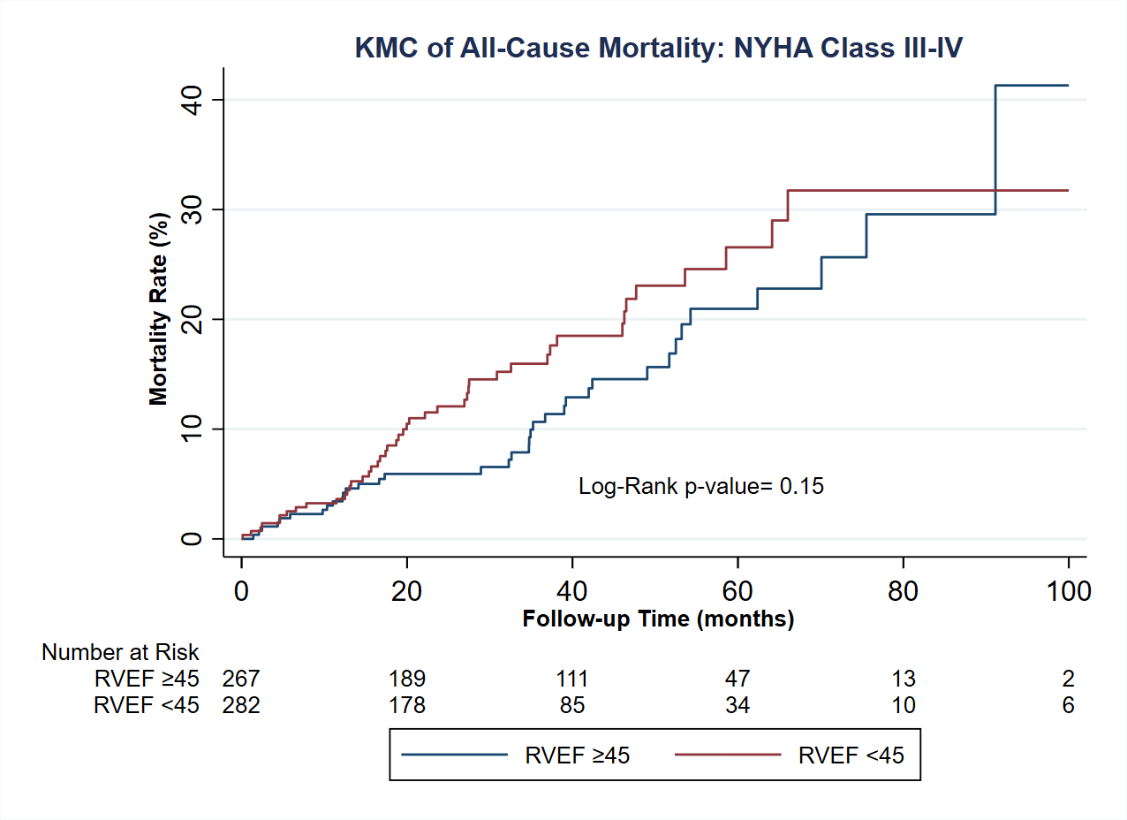
**

**Figure S3-b: Etiology of Cardiomyopathy (Ischemic vs. Non-ischemic)**
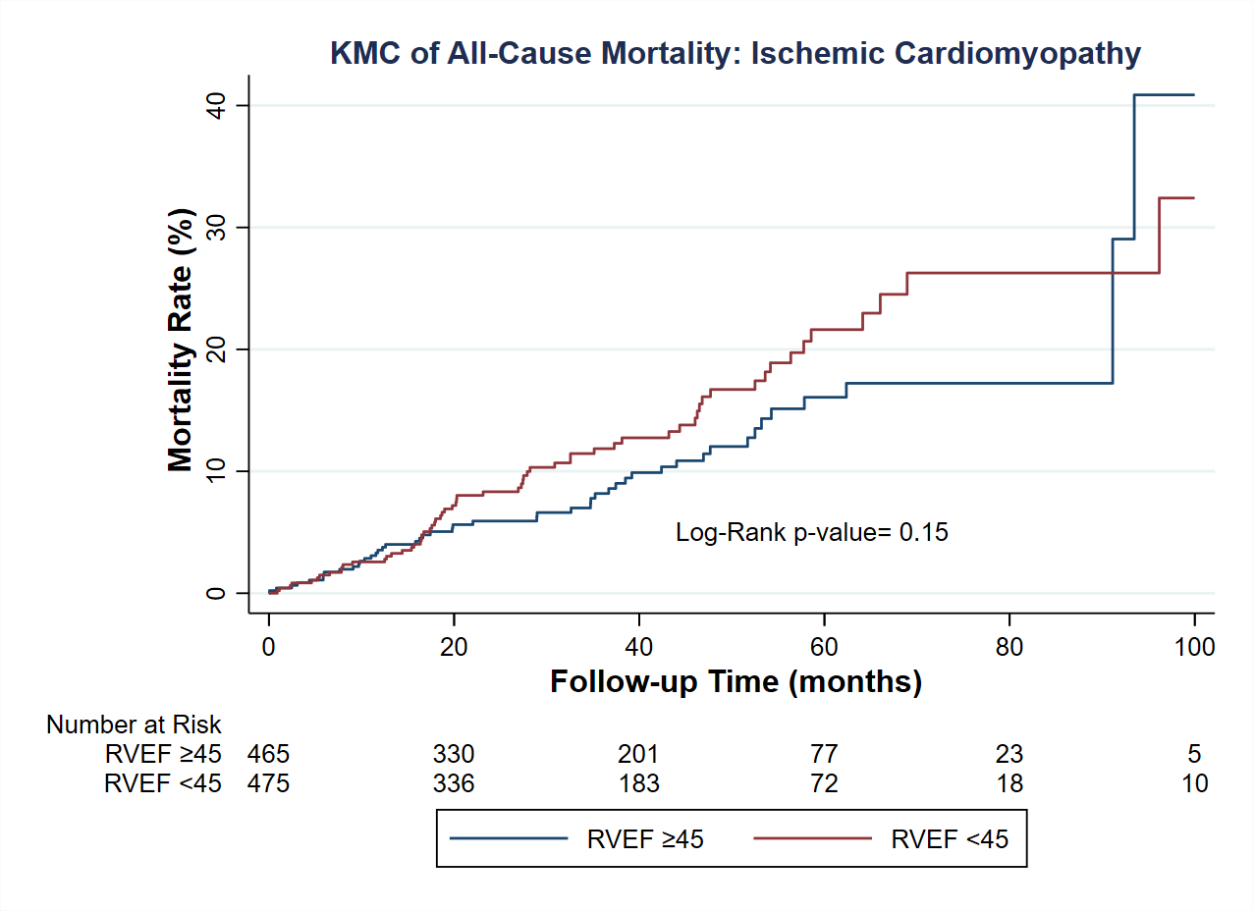


**
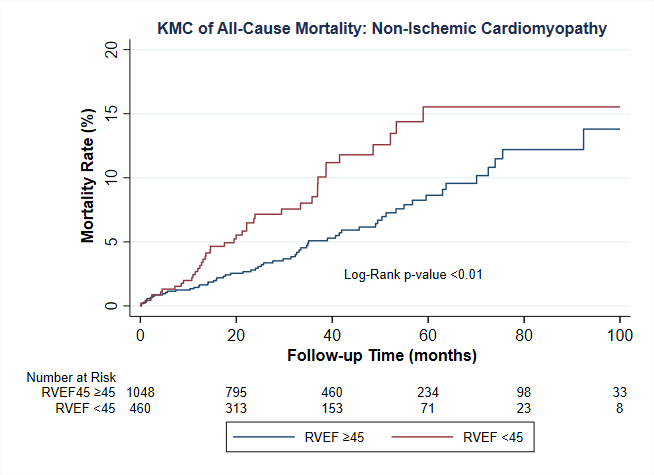
**

**Figure S3-c: Pulmonary Artery Systolic Pressure (PASP ≤35 vs. PASP >35 mmHg)**

**
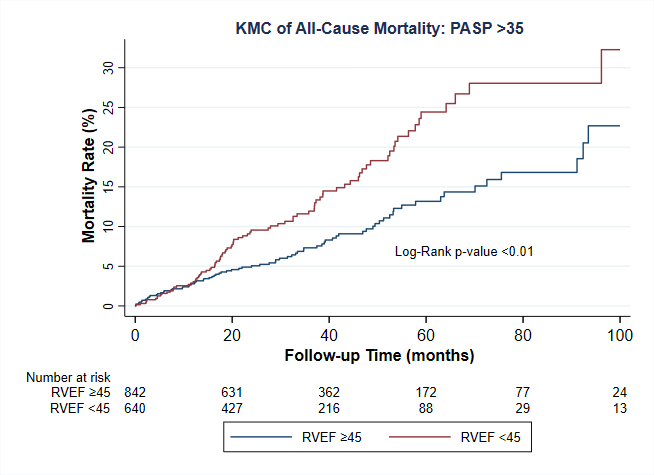
**

**
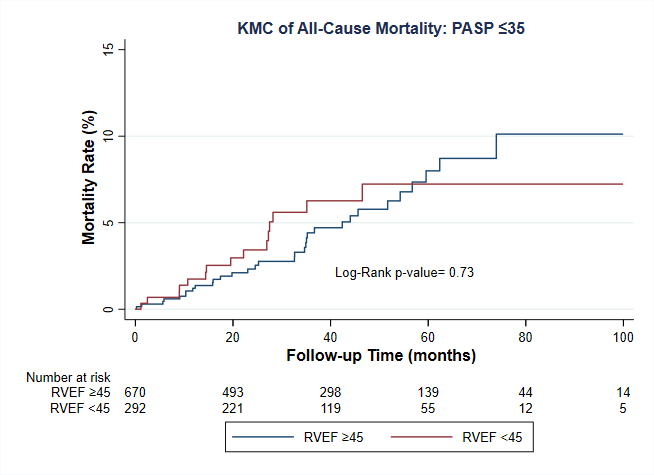
**

**Figure S3-d: Tricuspid Annular Plane Systolic Excursion (TAPSE <17 mm vs. TAPSE ≥17 mm)**

**
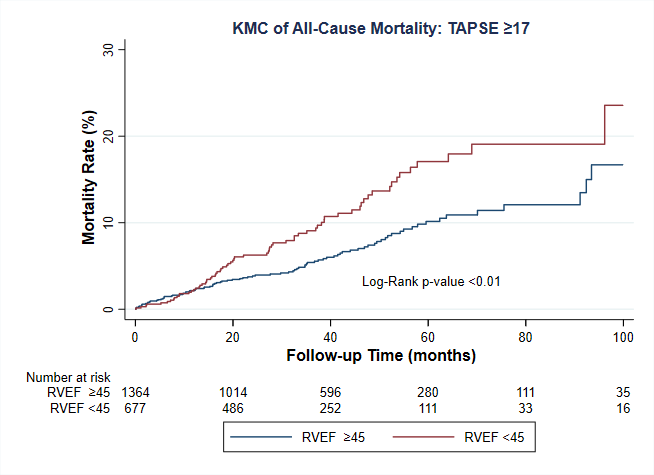
**

**
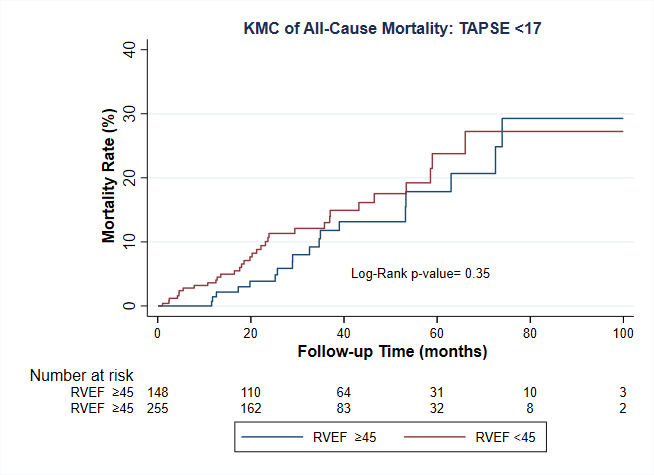
**

**Figure S3-e: Diabetes Mellitus**

**
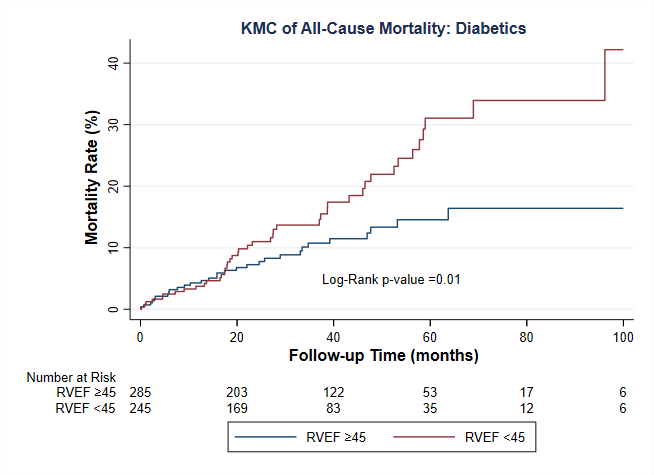
**

**
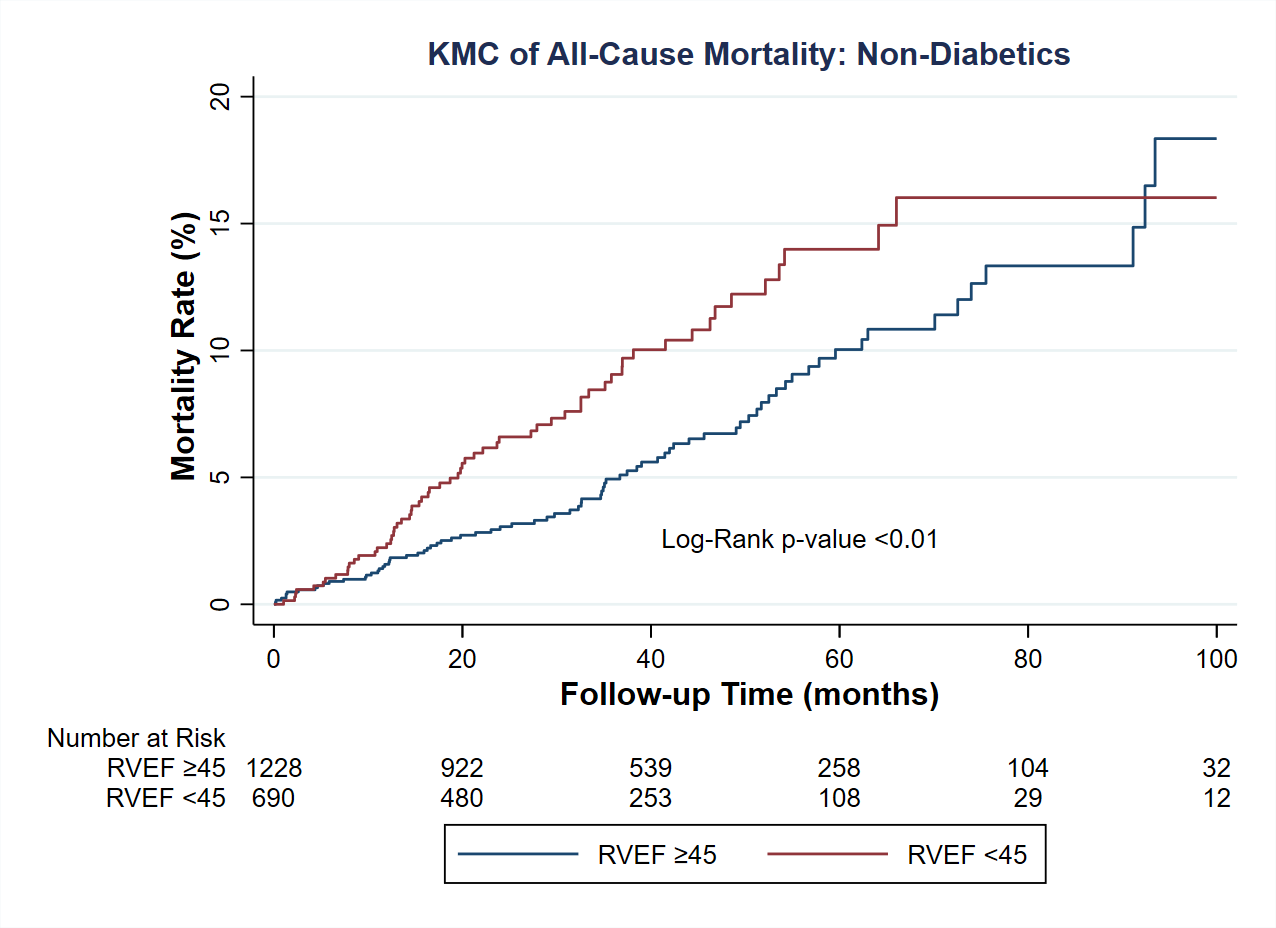
**

**Figure S3-f: Kidney injury (creatinine ≤ 1.5 mg/dL vs. >1.5 mg/dL).**

**
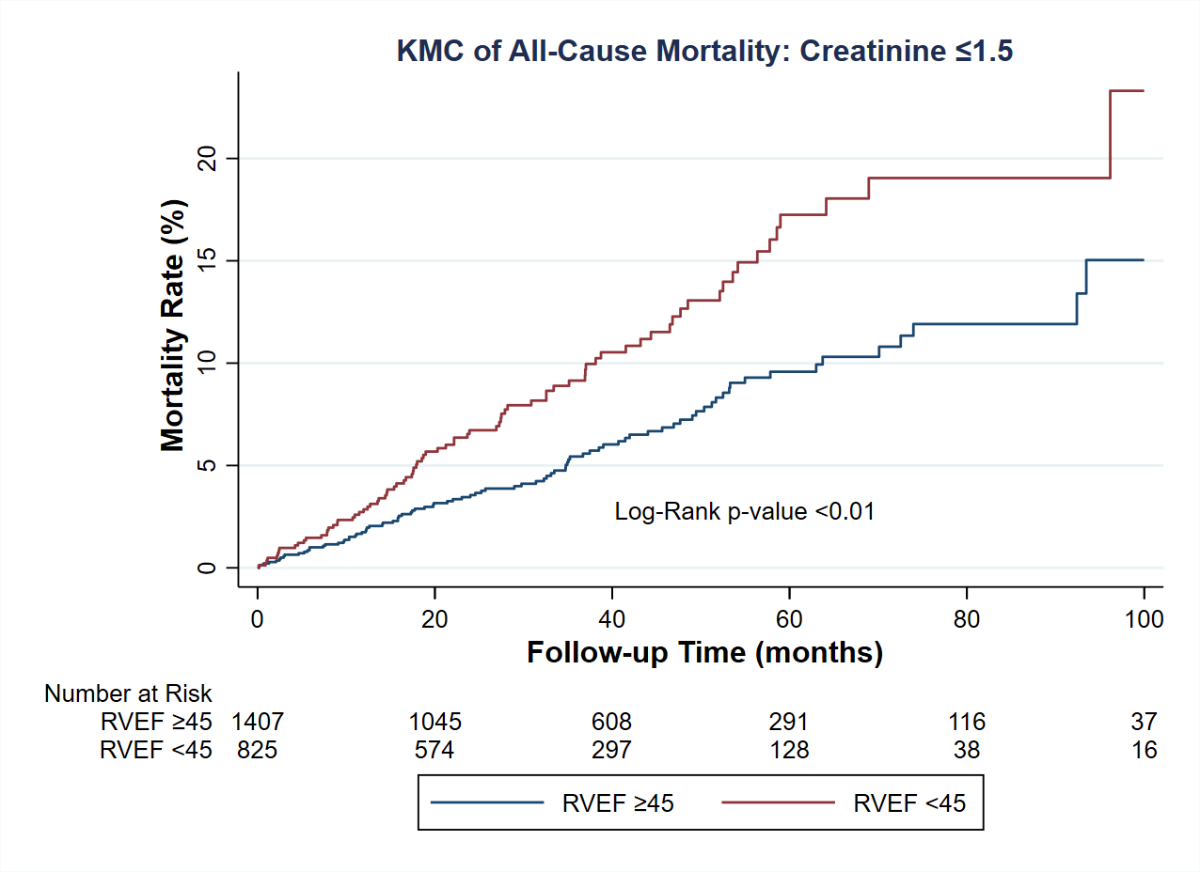
**

**
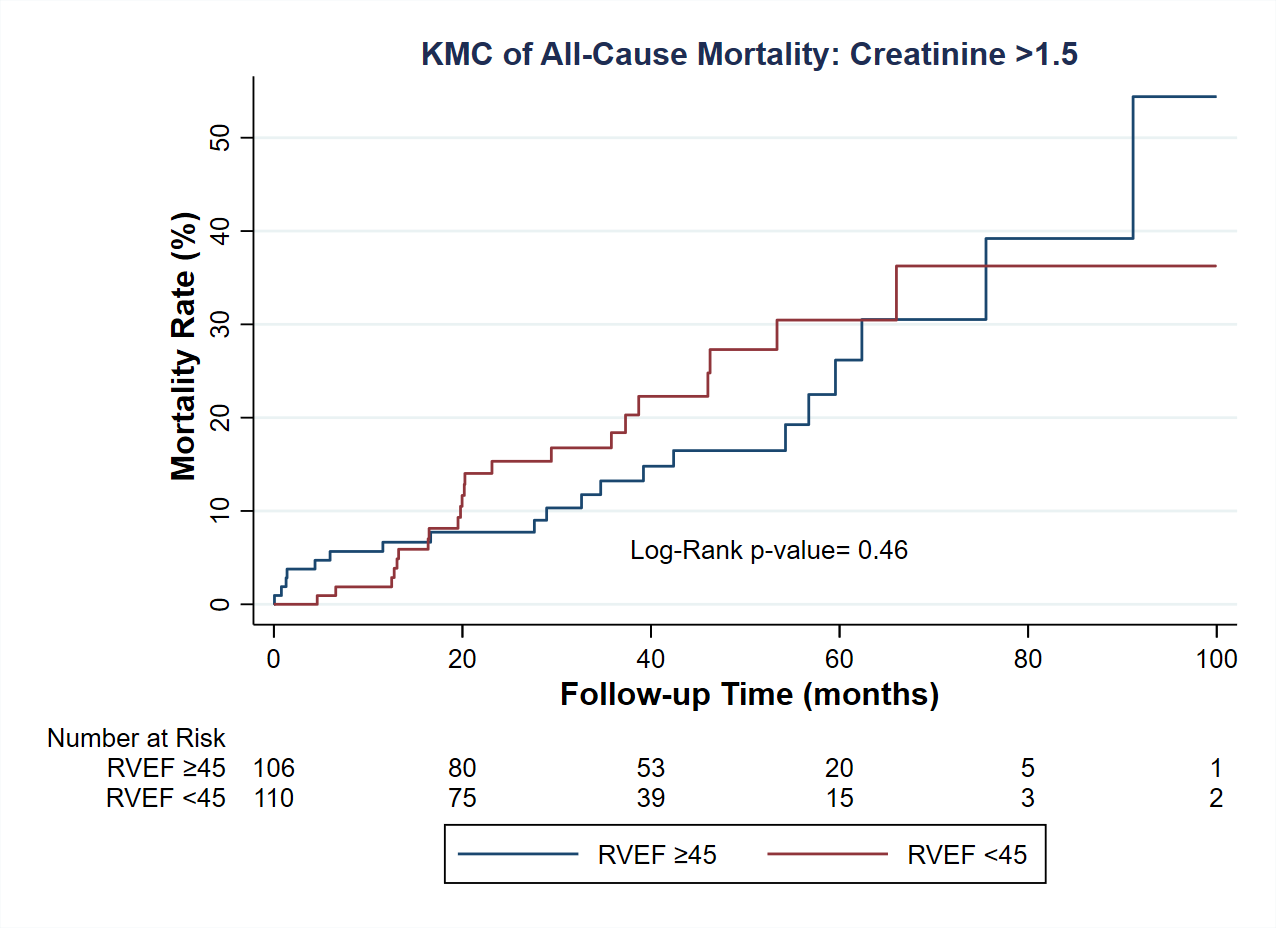
**

**Figure S3-g: LVEF (< 35% vs. ≥35%)**


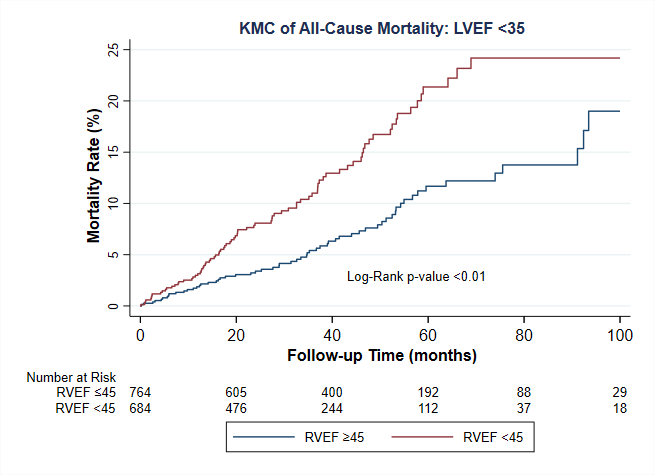


**
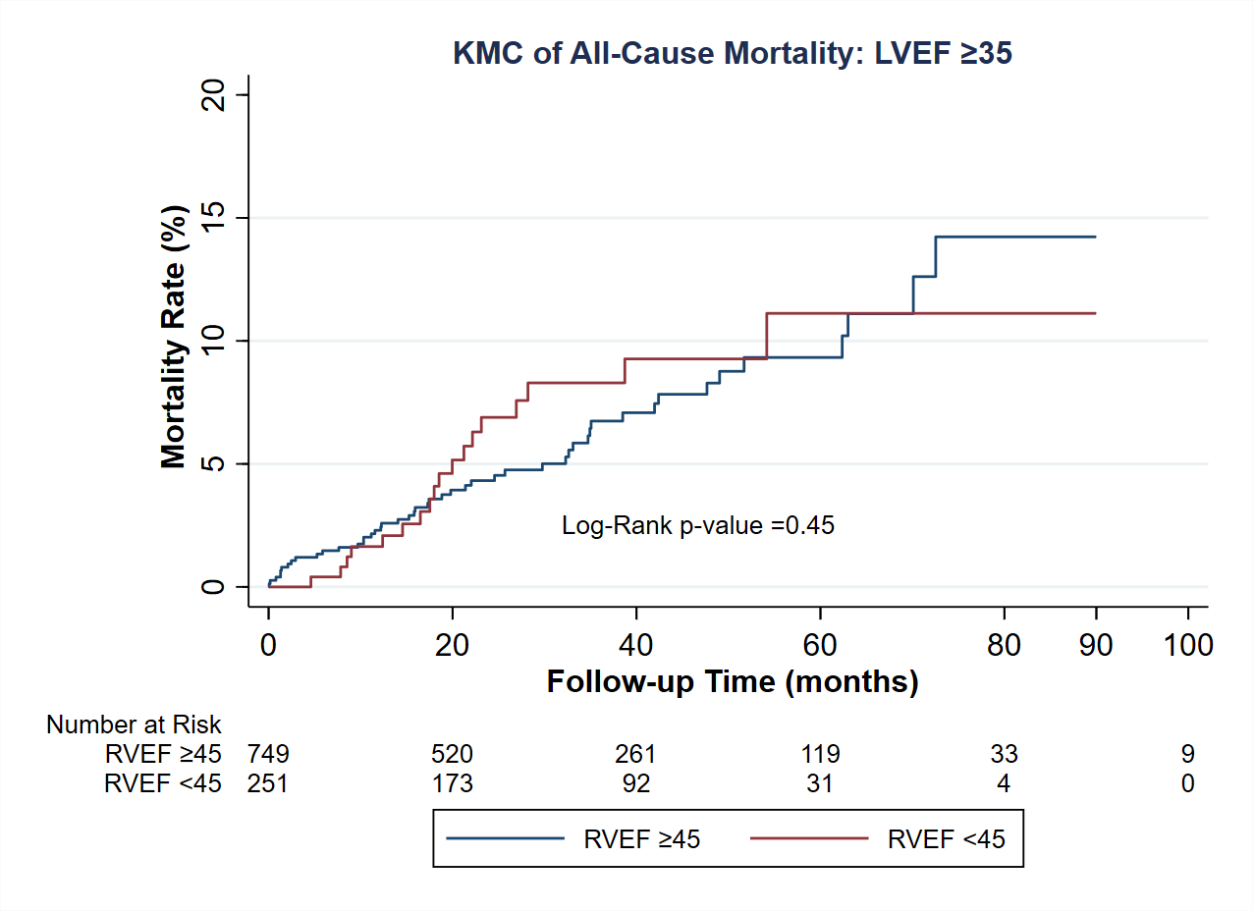
**

**Figure S4:** Correlation between right ventricular ejection fraction (RVEF) and the ratio of right ventricular systolic volume (RVSV) and right ventricular end-systolic volume (RVESV). The correlation plot includes data from 2,267 heart failure patients included in our study.

**
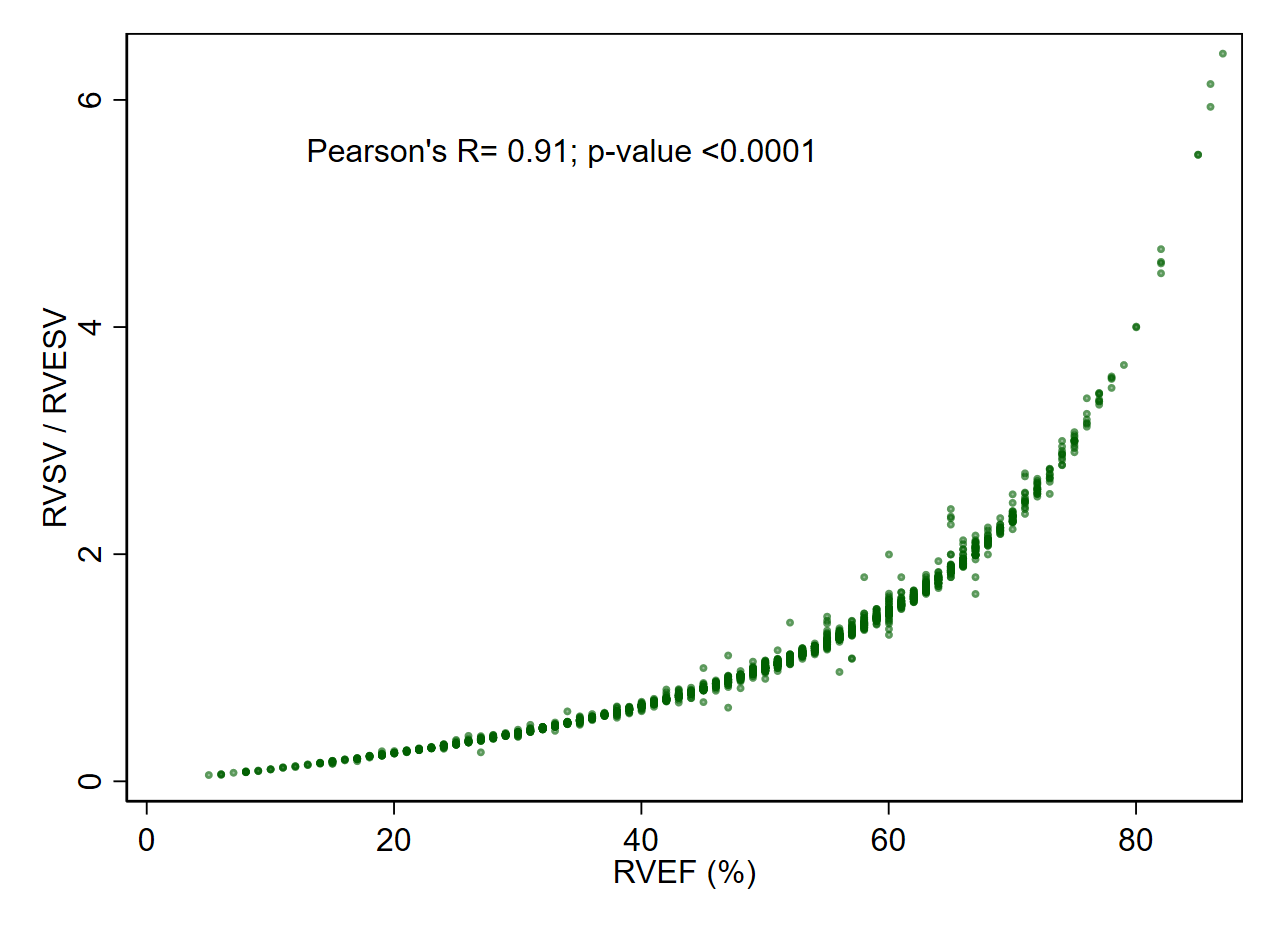
**

**Figure S5:** 10-fold cross validation method for regression model for:

1. All-cause mortality


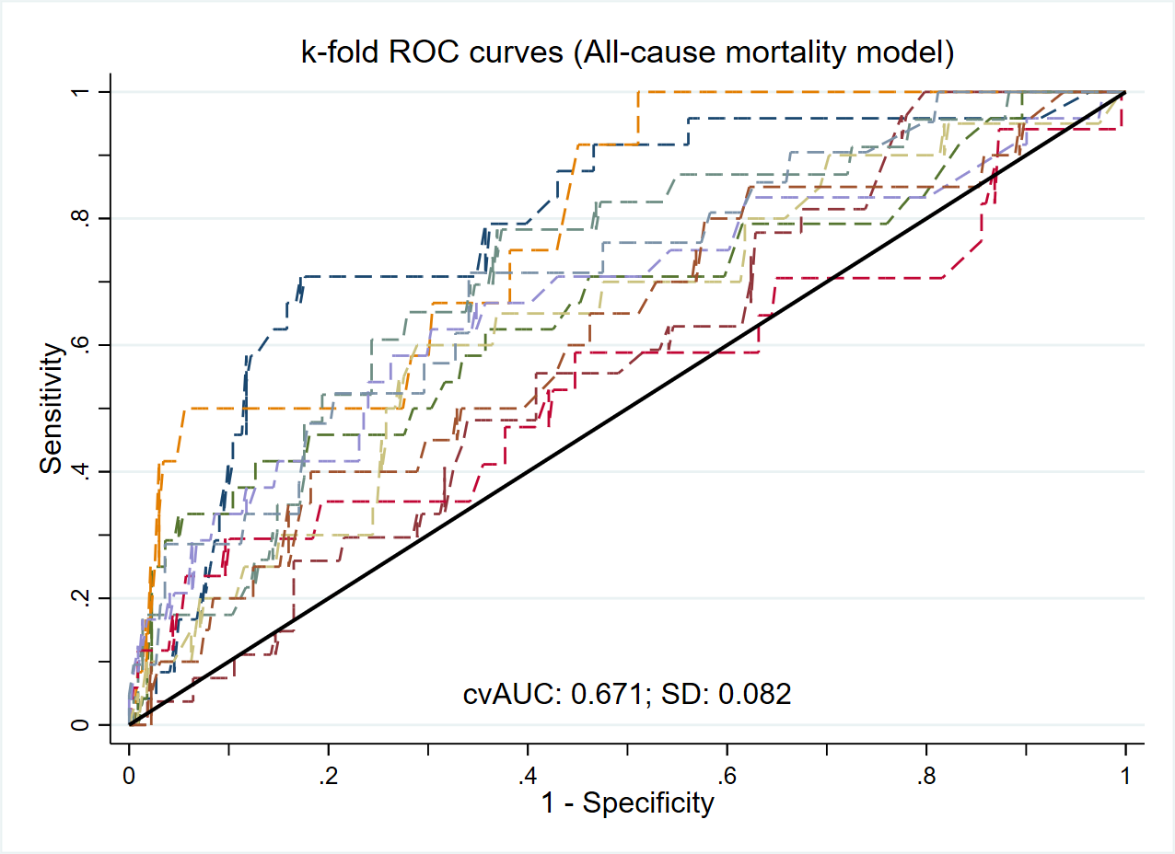


1. Composite outcome of all-cause mortality/HFH

**
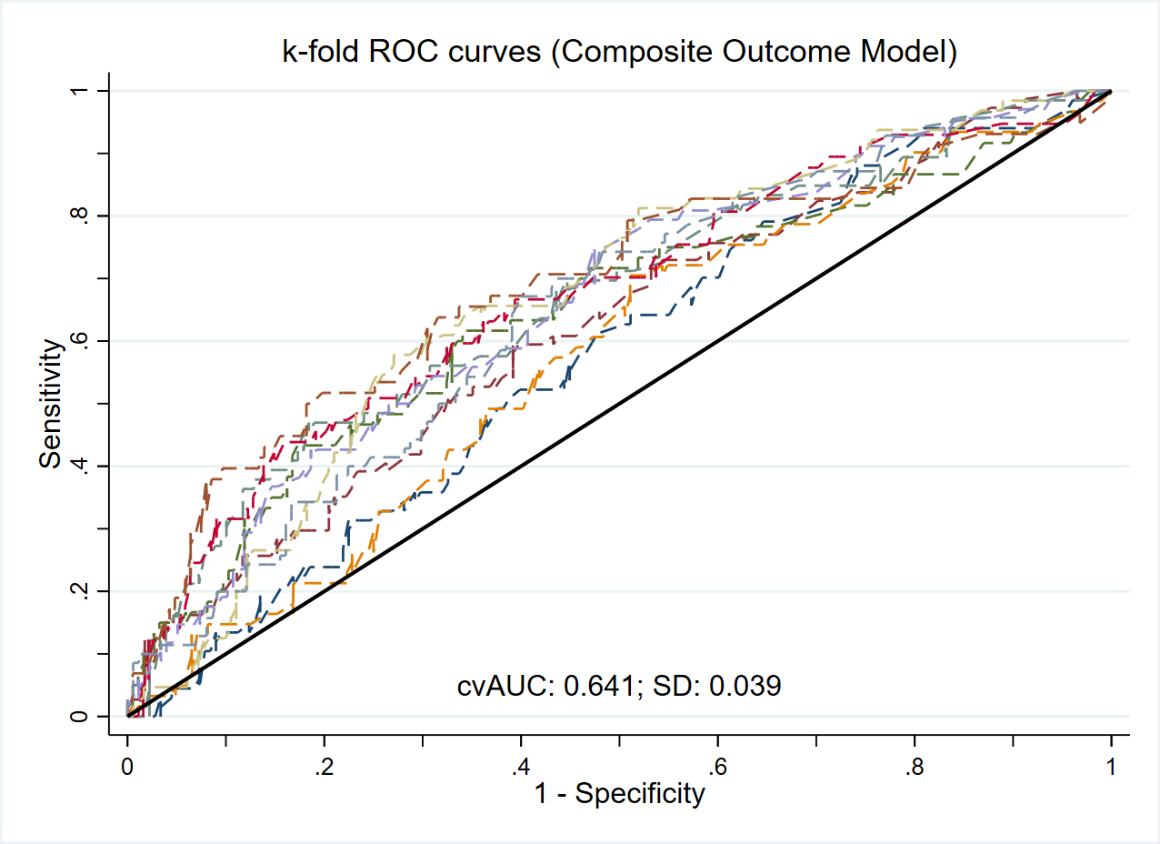
**
